# Predicting atrial tachycardia and major cardiovascular events in adults with unrepaired Ebstein's anomaly of the tricuspid valve

**DOI:** 10.1186/1532-429X-17-S1-Q76

**Published:** 2015-02-03

**Authors:** Riikka Rydman, Yumi Shiina, Michael A Gatzoulis, Dudley J Pennell, Philip J Kilner, Sonya V Babu-Narayan

**Affiliations:** 1 Clinical Physiology, Molecular medicine and surgery, Karolinska Institute, Stockholm, Sweden; 2 NIHR Cardiovascular Biomedical Research Unit, Royal Brompton, London, UK; 3 Cardiovascular centre, St Luke's International Hospital, Tokyo, Japan; 4 Imperial College London, National Heart and Lung Institute, London, UK

## Background

Patients with Ebstein's anomaly are at risk of tachyarrhythmia, congestive heart failure and sudden cardiac death. There are few data regarding the value of modern non-invasive diagnostic testing in prediction of outcomes. Therefore, we aimed to evaluate cardiovascular magnetic resonance (CMR) and compare it to known predictors in Ebstein's anomaly.

## Methods

Seventy-nine consecutive adult patients (aged 37±15 years) with unrepaired Ebstein's anomaly underwent CMR and were followed prospectively for a mean of 3.5±2.6years.The major adverse cardiovascular events were defined as sustained ventricular tachycardia(VT)/heart failure hospital admission/transplantation or death. Sub-group analysis of first-onset atrial tachyarrhythmia (AT) was performed.

## Results

Univariate predictors of the major adverse cardiovascular events (n=6 patients; 3 death, 3 VT) were New York Heart Association class >2 (Hazard ration (HR 7.66[95%CI 1.535-38.204],p=0.013), left ventricular (LV) ejection fraction (HR) 0.43[95%CI 0.245-0.742],p=0.003/5%), LV stroke volume (HR 0.69[95%CI 0.515-0.923],p=0.013/5mL) and right ventricular (RV) ejection fraction (HR 0.49[95%CI 0.276-0.856],p=0.012/5%); all remained significant even when tested solely for mortality. In all but one patient VT/death was preceded by AT and AT was predictive of major adverse cardiovascular events (HR 11.16[95%CI 1.299-95.813],p=0.028), emphasizing its role as an early marker of long-term adverse events. Freedom from first-onset AT (n=17 patients, 21.5%) declined during the follow-up. Univariate predictors for first-onset AT included RV ejection fraction (HR 0.65[95%CI 0.463-0.907],p=0.011/5%)], RV/LV end diastolic volume ratio (HR 1.65[95%CI 1.207-2.258],p=0.002) and apical tricuspid septal leaflet displacement indexed to LV length (HR 1.03[95%CI 1.002-1.068],p=0.040); when used together as a score, risk prediction was further enhanced (HR 4.84[95%CI 1.615-14.522],p=0.005).

## Conclusions

Ventricular functional impairment by CMR was highly predictive of mortality and VT in a large contemporary cohort of unrepaired adult patients with Ebstein's anomaly supporting the potential of CMR in risk stratification and clinical follow up. Furthermore, VT and death were preceded in almost all patients by presentation of AT suggesting that AT predicts long term adverse events early on in the disease progression. Onset of AT was best predicted by routinely measured CMR parameters feasible for clinical approach.

## Funding

Riikka Rydman was supported by the Swedish Society of Medicine, Swedish Heart-Lung Foundation and Swedish Society for Medical Research and by the Section of Clinical Physiology, Department of Molecular Medicine and Surgery, at Karolinska Institutet, Stockholm, Sweden. Sonya V. Babu-Narayan is supported by an Intermediate Clinical Research Fellowship from the British Heart Foundation (FS/11/38/28864). This project was supported by the National Institute for Health Research (NIHR) Cardiovascular Biomedical Research Unit of Royal Brompton and Harefield National Health Service (NHS) Foundation Trust and Imperial College London. This report is independent research by the NIHR Biomedical Research Unit Funding Scheme. The views expressed in this publication are those of the author(s) and not necessarily those of the NHS, the National Institute for Health Research or the Department of Health.

